# Nanomaterial-Based Therapy for Wound Healing

**DOI:** 10.3390/nano12040618

**Published:** 2022-02-12

**Authors:** Anamika Kushwaha, Lalit Goswami, Beom Soo Kim

**Affiliations:** Department of Chemical Engineering, Chungbuk National University, Cheongju 28644, Korea; kushwaha.anamika@gmail.com (A.K.); lalitgoswami660323@gmail.com (L.G.)

**Keywords:** chronic wounds, nanomedicine, chronic inflammation, angiogenesis, infection, mechanism, healing challenge

## Abstract

Poor wound healing affects millions of people globally, resulting in increased mortality rates and associated expenses. The three major complications associated with wounds are: (i) the lack of an appropriate environment to enable the cell migration, proliferation, and angiogenesis; (ii) the microbial infection; (iii) unstable and protracted inflammation. Unfortunately, existing therapeutic methods have not solved these primary problems completely, and, thus, they have an inadequate medical accomplishment. Over the years, the integration of the remarkable properties of nanomaterials into wound healing has produced significant results. Nanomaterials can stimulate numerous cellular and molecular processes that aid in the wound microenvironment via antimicrobial, anti-inflammatory, and angiogenic effects, possibly changing the milieu from nonhealing to healing. The present article highlights the mechanism and pathophysiology of wound healing. Further, it discusses the current findings concerning the prospects and challenges of nanomaterial usage in the management of chronic wounds.

## 1. Introduction

The human body is covered by the skin, which is the largest organ. Approximately 2 m^2^ of the adult surface area is skin. The skin acts as a defensive barrier between the humanoid body and the exterior milieu. Thus, it functions as moisturizer, sensory perception, temperature control, humoral equilibrium maintenance, and resistance to external pathogens [1]. With prolonged exposure, the skin tolerates the impact of numerous exterior stimuli. Injuries triggered by the destruction of the skin’s integrity led to ailments. The most recurrent injuries are burns, medical incisions, contusions, cuts, and scratches caused by trauma. The self-healing property of an organism results in wounds healing within three months, depending upon the injury’s severity. However, an uncontrolled infection transforms an acute infection into a chronic one that lasts for months or even years [2].

Recently, chronic ailments such as vascular dysfunction, obesity, and diabetes have increased remarkably, causing a surge in patients suffering from chronic wounds. The diabetic patient has a 15–25% risk of diabetic chronic abscesses [3]. Moreover, some communicable skin ailments, for instance, malignant skin tumors, sporotrichosis, autoimmune skin diseases, dermatomyositis, and physical skin disease, can make patients vulnerable to chronic wounds [4]. The hypodermic tissue of chronic and nonhealing wounds is bared to the exterior environment for an extended period, predisposing patients to bleed and osteomyelitis and thereby posing the risk of death for patients in severe conditions. The occurrence of chronic infection decreases the quality of life in patients, upsurges their financial burden, and leads to severe mental and psychosocial complications. In the United States, for example, millions of patients are suffering from bone or skin imperfections. The treatment sums for these defects are USD 39 billion and USD 75 billion annually, respectively [2]. In addition, prolonged medicinal resources have caused a burden on the healthcare system.

Wound healing is continuously becoming more complex for clinicians, and novel materials and approaches are instantly desirable. Substantial developments in nanotechnology, principally in nanochemistry and nanomanufacturing, have revolutionized the pharmaceutical and biotechnology industries. Nanomaterials (with at least one dimension below 100 nm) display distinct physicochemical properties owing to their distinct structure, leading to small size, surface, and macroscopic quantum tunneling effects. Recently, nanomaterials have also been extensively utilized in wound healing owing to their better adsorption capacity, antimicrobial properties, and drug loading [5].

Wound dressings act as impermanent skin alternates and play an essential part in hemostasis, infection control, and wound closure. Several dressing materials have been explored for many years. Traditionally, wound dressings, such as gauze and bandages, were used for treating skin defects [6]. The model dressing must simulate with the extracellular matrix (ECM) for a wet milieu, possess antimicrobial properties, and encourage the proliferation of cells and angiogenesis, therefore, needing unique constituents with remarkable properties. The huge demand in the market for such resources has enhanced the growth of nanomaterial dressings [7]. Presently, novel nanomaterial-based bandages, such as hydrogels, nanofibers, and films, are now being extensively utilized. Interestingly, by 2021, the market for such materials is predicted to surpass USD 20.4 billion globally [8,9].

Even though a growing number of novel nanomaterials have been reported for their utilization in wound healing, the mechanisms have not been thoroughly summarized. Henceforth, in the present review, we explored recent nanomaterial applications, possible mechanisms, and potential toxicity to aid in wound healing from diverse facets. Prominently, we present the limits of the current application of nanomaterials in the medical and mechanistic studies of wound healing and provide solutions and innovative research concepts that can become future directions of investigation/research.

## 2. Physiology of Wound Healing

**Figure 1:**
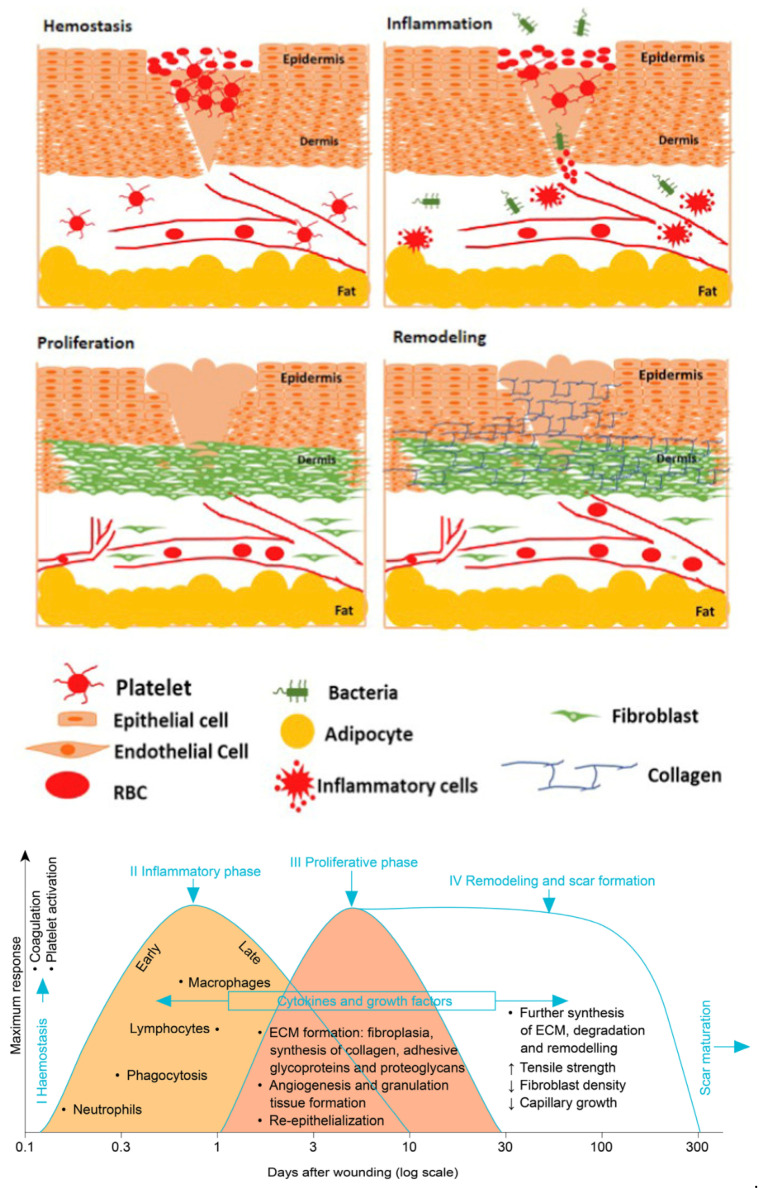
Four stages of cutaneous wound healing (upper) [10] (Reprinted with permission from Ref. [10]. Copyright Elsevier, 2020) and corresponding timelines (lower) [11] (Reprinted with permission from Ref. [11]).

The restoration of skin is a complicated physiological procedure, which involves the complex organization of numerous diverse cell types, chemokines, and various growth factors in a chronological manner. Conventionally, the wound healing process is categorized into four phases: (a) hemostasis, (b) inflammation, (c) proliferation, and (d) remodeling (Figure 1).

### 2.1. Hemostasis Phase

Initially, hemostasis is the primary response to injury, stopping bleeding and abating hemorrhaging followed by vascular injury. Hemostasis involves three stages; vasoconstriction followed by primary hemostasis and then secondary hemostasis, which is attained through fast, synchronized, and mechanistically interrelated routes [11,12,13,14]. Table 1 represents the function of various biomolecules involved in wound healing. In addition, they overlay the subsequent phase of the wound healing stage through the stimulation of epithelial cells, employing fibroblasts for collagen deposition and encouraging the restoration of injured tissue [15,16].

### 2.2. Inflammation Phase

The inflammation arises instantly after the injury and frequently lasts for up to 3 days. After the strong vasoconstriction of early hemostasis, the degranulation of platelets triggers a complement cascade and produces strong complement peptides, which leads to the discharge of histamine, serotonin, proteases, and additional cellular intermediaries from basophils and mast cells. It causes vasodilation and amplified vascular permeability and blood flow, developing inflammation and heat [26,27,28].

After 3–4 days of wound development, macrophages remove exhausted neutrophils via efferocytosis, averting a nonspecific breakdown of the tissue and perseverance of inflammation [12,29].

### 2.3. Proliferation Phase

Four days after wound development, a proliferation stage begins and persists for ~21 days in chronic wounds. However, the period is also influenced by the wound size and patient health. The proliferation stage is categorized primarily by granulation of tissue, wound reduction, and angiogenesis [30]. During granulation, angiogenesis plays an essential role, and the tissue growth is activated during hemostatic plug due to the generation of TGF-β, PDGF, and FGF via the platelets. Further, the granulation tissue developed during inflammation functions as a primary tissue and is eventually substitutes the clot at the injured site [27].

The re-epithelialization process is supported by the granulation tissue, where several epithelial cells frequently travel across new tissues resulting in the generation of barriers amid the wounded area and the environment [31,32]. Granulation tissue gathers cellular–ECM interactions, and biomolecules are released along with growth factors and mechanosensory signals stimulating the differentiation of fibroblasts into myofibroblasts that aids in drawing wound boundaries together via wound contraction [15].

### 2.4. Remodeling Phase

The last stage of wound healing is maturation and remodeling. This phase starts about three weeks post wound development and can take one year or even more, based on the type of wound, resulting in the natural epithelium growth and scar tissue maturation [11]. This phase is simply the balance between synthesis, deposition, and degradation. Several myofibroblasts, endothelial cells, and macrophages help in wound remodeling. Fibroblasts help in crosslinking collagen resulting in the collagen realignment into organized grids that upsurge the tensile strength of the tissue, accomplishing about 80% of unwounded skin [33]. Despite its seemingly important role in re-epithelialization, the absence of this growth factor does not hinder wound healing. Finally, there is a completely matured scar with reduced vascularity. Therefore, wound restoration is complex and contains an appropriate arrangement of stages; any failure in the synchrony stages will result in chronic wounds [15].

## 3. Pathophysiology of Wound Healing

In general, skin establishes a high capacity for restoration that is controlled by the efficient and arranged order of cellular and molecular processes. Furthermore, a disturbance in the normal healing procedure may entirely stop wound healing, causing chronic wounds, such as arterial ulcers, nonhealing surgical wounds, foot ulcers, and pressure sores [15]. Conditions related to abnormal wound healing comprise numerous interconnected aspects such as cell senescence/apoptosis, protracted inflammation, production of matrix metalloproteinase (MMP), matrix degradation, infection, systemic reasons including nutritional conditions of patient, strain, and other chronic comorbid [34,35].

The acute wound microenvironment establishes a well-ordered ECM and a matrix synthesis rate that surpasses its breakdown. Normal injuries are typically categorized by a low bacterial load (as the microbial infection is controlled by immune cells) and quickly heal with propagation and restoration. The imbalance in the phases results in chronic, nonhealing wounds [36,37,38]. Here, chronic wounds are described as those that have not progressed due to an organized and appropriate process to attain the usual anatomic and functional integrity [39,40]. Several investigators stated that 6–8 weeks are suitable for wound healing; beyond that, the wound should be considered a chronic/nonhealing wound. The delayed recovery can be due to wound infection, the perseverance of foreign particles or microbial proteins, chronic irritation and shock, and ischemia [41,42,43,44,45,46].

**Figure 2:**
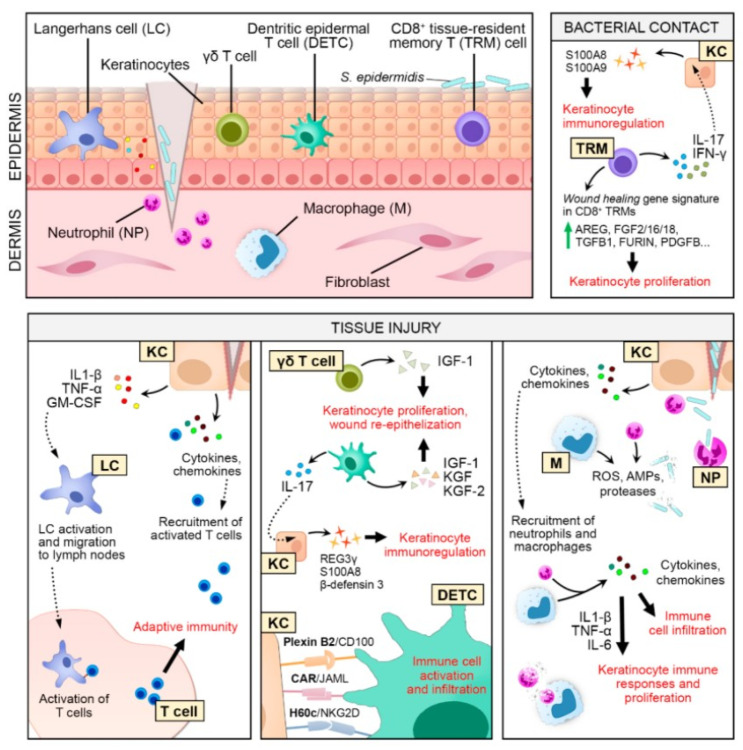
Graphical representation of cellular and molecular mechanisms during a nonhealing chronic wound (Reprinted with permission from Ref. [38]).

The continuous interleukins (ILs) and inflammatory cytokine expression and overexpression cause the imbalance in vital proteases, growth factors, and cytokines, which results in excessive degradation, proteolysis, and inadequate accessibility of crucial receptors, growth factors, and ECM. Cells during acute wound healing are different (functionally and phenotypically) from that of chronic nonhealing wounds. The proliferation and movement of fibroblast cells are averted and do not respond to growth factors [36]. Some immune cells responsible for bactericidal and phagocytic activity become disrupted, thus accumulating necrotic debris near the edge of the wound [15]. Chronic wounds also lead to biofilm formation compared to acute injuries [37]. Damaged vascularization and an inadequate transfer of oxygen and nutrients to the cells stimulate avascular necrosis, which later causes bacterial growth and the formation of a biofilm. Subsequently, biofilm formations trigger the inflammation, thereby averting the deposition of ECM and wound epithelialization. Figure 2 represents the molecular mechanisms of chronic wounds. The wound site in chronic wounds is larger than the noticeable lesion, which is caused due to the pathological injury to tissues around the wound where the integrity of the skin is damaged [47,48,49,50,51,52]. The tissues around and underneath the chronic wounds are similarly influenced by the primary abnormality causing ulceration [42,53,54,55,56,57,58]. The factors (local and systemic) influencing wound recovery include insufficient blood supply, the existence of foreign particles that hinder the tissue repair, the constant presence of microbes and infection, wound dehiscence, impairment of venous drainage, decreased macrophage activity, and increased MMP levels [59,60,61,62,63,64,65].

## 4. Significance of Nanoparticles in Wound Healing

Nonetheless, wound healing is a challenging issue making wound management crucial. Nanotechnology delivers several novel approaches for regenerative medicine. Recently, numerous biocompatible self-assembling nanoparticles (NPs) were developed [66,67,68,69,70]. NPs boost deferred wound recovery and injury treatment. The metal NPs, such as zinc oxide, gold, and silver, have displayed favorable properties, such as less in vivo toxicity and bacteriostatic and bactericidal activities [15,71,72,73,74]. Typically, NPs are in the nm size range, and the usage of nanomaterials in contemporary treatment is a rapidly growing area. It involves the expansion of materials in the range of nanometers or in the molecular range [75]. The decrease in the size of a material to a nanoscale leads to a surge in surface area and surface area to volume ratio, which results in progressive physiochemical properties.

### 4.1. Metal and Metal Oxide NPs

NPs based on metal are extensively applied in biomedicine owing to their benefits, such as easy synthesis with defined shapes and sizes, facile functionalization of the surface, enhanced biocompatibility, and superior physicochemical properties [76]. The most extensively used NPs in the biomedical area are gold and silver. Gold NPs are broadly considered in biosensing due to their optical properties and for drug delivery as a nanocarrier [77]. Silver NPs are primarily used as antimicrobial agents and anticancer activity [78]. Three hypothetical mechanisms have been demonstrated for their antibacterial activity: (i) the perviousness of microbes will be altered due to the NP accumulation on the microbial membrane therefore causing the release of membrane proteins, lipopolysaccharides, and intracellular biomolecules; (ii) the generation of reactive oxygen species (ROS) via NPs, instigating oxidative impairment to cells; (iii) NPs are metabolized by the microbes, causing a reduction of intracellular ATP and a disturbance in the replication of DNA [79,80]. Metal and metal oxide nanoparticles display a virtuous bactericidal capability towards the existing drug-resistant strains [81]. However, it has also been stated that microbes would advance the capacity to escape these NPs when used on a pilot scale [82], and this area should be explored in future studies.

#### 4.1.1. Silver NPs

Silver is a bactericidal agent and is generally used for the treatment of blisters, wound disease, and abscesses. For instance, silver nitrate is still used for the treatment of nonhealing chronic disease. For effective drug distribution, diverse silver coatings in wound bandages are accessible nowadays, which play a significant role during chronic wound ailment [83,84]. Dressing based on silver nanoparticles (AgNPs) does not lead to a complication, even when applied for a protracted period. The combination of collagen and AgNPs displays robust antibacterial activity, thus making it an appropriate element during wound covering [85]. Metal nanoparticles as a single conjugate have been known to carry efficient properties for wound healing. In view of their remarkable antimicrobial properties, AgNPs have been used in various types of wound coverings, for instance, chitosan [86], poly(vinyl alcohol)/sodium alginate hydrogels [87], and plasma-treated electrospun polycaprolactone scaffold [88]. The wound coverings loaded with AgNPs developed from diverse biocompatible polymers showed virtuous repressive activity against *Staphylococcus aureus, E. coli, S. epidermidis,* and *Salmonella typhimurium*.

The combination of collagen/chitosan scaffolds with AgNPs (AgNP–collagen/chitosan hybrid scaffolds) was observed to be effective against bacterial infection in the skin [89]. The formation of biofilms in the infected area may obstruct the diffusion of therapeutic agents that significantly reduce the antiseptic effectiveness [90]. It was found that within 1 h, AgNPs (~20 nm) could enter an *E. coli* (40 μm) biofilm, and AgNPs dispersing inside the biofilm can then dissolve, and Ag^+^ is released, resulting in enhanced antibacterial activity. Nevertheless, AgNP antibacterial efficiency toward biofilm will be significantly decreased owing to their propensity to aggregate [91]. In addition, AgNPs are comparatively lethal to humanoid cells, which is one of the foremost hindrances for their implication in wound healing.

#### 4.1.2. Copper NPs

In wound healing, copper NPs (CuNPs) have been extensively investigated due to their remarkable antibacterial property and observed to suppress a wide spectrum of bacterial strains [92]. CuNPs release Cu^2+^, which causes disruption in cell walls and membranes by hardening the protein structure or altering the enzyme function. The antibacterial efficiency of CuNPs displays size- and concentration-dependent activity [93]. For instance, at high concentrations, CuNPs showed complete inhibition of *E. coli* [94]. The CuNPs adhered to the bacteria and showed penetration ability to the cell membrane. First, released Cu^2+^ damaged the cell walls of bacteria, followed by cytoplasm degradation and finally bacterial death. Earlier, the various sizes and concentrations of CuNPs were investigated for the treatment of bacteria in the wound healing procedure [93]. It was found that CuNPs with 40 nm and 80 nm sizes and 10 μM and 1 μM were not lethal to the skin.

Additionally, Cu atoms showed a catalytic activity similar to Fenton chemistry. During recycling redox reactions amid Cu^+^ and Cu^2+^, hydrogen peroxide was released, which resulted in damage to the bacterial cytoplasmic membrane leading to bacterial death [95]. After CuNPs were taken up by bacteria, ROS was generated in the bacteria, and it was examined that CuNPs augmented the ROS level by 2.5 fold compared to the control [96]. The ROS overproduction will eventually kill the bacteria via cellular lipid and protein peroxidation and DNA degradation. Moreover, CuNPs can help avert the development of *Pseudomonas aeruginosa* biofilm at more budding phases [97]. CuNPs (100 ng/mL) displayed 94% *Pseudomonas* biofilm inhibition, representing a higher antimicrobial efficiency than AgNPs [97]. CuNP-coated wound dressing showed the rapid and efficient killing of *Acinetobacter* in skin wounds [98]. CuNPs stimulate angiogenesis by affecting the expression of the hypoxia-inducible factor (HIF-1a) and regulation of the secretion of VEGF, thereby promoting wound healing [97].

#### 4.1.3. Gold NPs

Biocompatible gold nanoparticles (AuNPs) are widely used in wound healing, tissue regeneration, and targeted drug delivery [99]. Unlike Ag, AuNPs by themselves do not show any antimicrobial activity. Therefore, AuNPs should be combined with the other biomolecules to be utilized efficiently in the biomedical field. The crosslinking of AuNPs with collagen makes it easy to be combined with other biomolecules such as peptides, growth factors, polysaccharides, and cell adhesion molecules by impending at the surface of Au without altering the collagen structure [100]. These altered AuNPs displayed properties such as biodegradability and biocompatibility, and henceforth they might be utilized extensively in wound healing.

Likewise, chitosan and gelatin might further be included easily with AuNPs, which offer innocuous and beneficial effects in increasing wound healing [101]. Au can be coupled to other current NPs or antimicrobial drugs, thus enhancing their effectiveness toward killing microbes. For instance, vancomycin-conjugated AuNPs improved the activity of vancomycin (50 fold) against vancomycin-resistant enterococci and presented substantial activity against *E. coli* [102]. AuNPs can be coupled with pathogen-specific antibodies and photosensitizing molecules for photothermal therapy [103] and photodynamic therapy [104], respectively. For example, AuNP-conjugated photosensitizer exhibited enhanced antifungal activity in mice wounds against *Candida albicans* [105]. Recently, the topical application of AuNPs to cutaneal wounds in rats displayed improved recovery characterized by augmented re-epithelialization, granulation tissue formation, ECM deposition, and collagen fibres content [106]. These differences are mainly found in the initial healing phases and decrease the overall healing duration. AuNP and AgNP monotherapy were equated for in vitro wound healing utilizing rats; the AuNP therapy presented better free radical scavenging activity and improved wound healing [106].

With the conjugation of AuNPs with polymers or stem cells, AuNP wound healing activity was significantly enhanced [77,107]. Chitosan–AuNP conjugate upsurged the AuNP free radical scavenging activity numerous times and offered improved biocompatibility. A study on a rat surgical wound model exhibited that chitosan–AuNPs significantly enhanced the hemostasis, formation of epithelial tissue with a high healing rate, and closure of wounds in comparison to the standard chitosan and Tegaderm bandage alone [108]. In another investigation in rats, AuNPs were conjugated with human cryopreserved fibroblasts and applied on the surface of burn wounds. The wounds showed an improved recovery rate, decreased inflammation, and enhanced collagen deposition [107].

#### 4.1.4. Zinc Oxide NPs

In cosmetics and fillings in therapeutic products, zinc oxide NPs (ZnO NPs) have been widely utilized due to their biosafety, biocompatibility, and bactericidal properties [15]. ZnO NPs are adsorbed and accumulated on the bacterial cell surface and cytoplasm, respectively, and hinder the cell membrane leading to the death of the bacteria. Earlier investigations revealed that the ZnO NP size plays a crucial role in eliminating a wide range of pathogenic microbes [109]. For instance, ZnO NPs measuring 8 nm (1 mM) showed >95% growth inhibition of the *S. aureus* strain RN6390, but NPs measuring size 50–70 nm (5 mM) displayed only 40–50% bactericidal activity [109]. Likewise, a confocal laser scanning microscopy study demonstrated that smaller-sized ZnO NPs resulted in high inhibition of *S. aureus*, signifying the NP size-dependent antibacterial activity. This was possibly because of the higher amount of ZnO NP deposition on the bacterial surface owing to the smaller sizes, which eventually accumulate in the cytoplasm and cell membrane, causing cell death [109].

ZnO NP aqueous suspensions generate a high quantity of ROS, displaying antibacterial activity [110]. It was also observed that ZnO NPs could significantly kill *Mycobacterium* through direct contact and high ROS generation [110]. In addition, ZnO NPs could prevent *P. aeruginosa* and *S. aureus* biofilm formation in a dose-dependent manner [110]. The embedded ZnO NPs in chitosan hydrogel [111], collagen dressing [112], or cellulose sheets [113] showed both antibacterial and tissue regeneration activity, which made them suitable to decrease the risk of infections during wound healing [112,114].

### 4.2. Peptide Nanostructures

Peptide nanoparticles (PNPs) are formed via self-assembly and molecular chemistry methods. PNPs are a developing area in synthetic biology for various applications in the biomedical field, primarily for targeted drug delivery. PNPs are employed in biological drugs and are beneficial in interpreting cell signaling [115]. The self-assembled peptide frameworks closely impersonate the natural ECM and might further be improved functionally to upsurge their contact with other cells [116]. For instance, peptide hydrogel displays high biocompatibility and cytocompatibility in biological systems and numerous mammalian cells [117]. The synthesized peptides are turned into fibrils and are thus transformed into hydrogels and are activated in precise cell culture media without change in their viability [118].

Few newly formed peptide hydrogels showed a stimulative part in the in vitro studies, with the capability to aid in the attachment of cells and initiate the progenitor cell differentiation of liver into hepatocytes, helping significantly in liver tissue revival [119]. Several studies exhibited that peptide hydrogels delivery could start the endogenous endothelial cell survival by improving the neighboring microenvironment [120]. With the incorporation of peptide amphiphile into the cell adhesion epitope, the developed hydrogels display a growing cell-responsive environment and provide an appropriate environment for dental stem cell growth [121].

Similarly, a novel approach to combining various hyaluronic acids together to create a self-sealing pouch has recently been established. Encapsulated human mesenchymal stem cells (MSCs) were filled into this pouch to transport MSCs to desired places for tissue regeneration [122]. Recently, studies demonstrated that self-assembled conjugated peptides encouraged chondrocyte revival and functional repossession in bone damage [123]. These peptide hydrogels showed a positive effect in the regeneration of tissue, in contrast to contemporary natural biomaterials. Initially, these self-assembled nanostructures deliver a suitable condition for cell development and differentiation during tissue regeneration. Second, amino acid-derived PNPs retain better biodegradability and biocompatibility. Finally, these peptide sequences can be tailor made and combined into nanostructures for desired target sites depending on the requirement. Moreover, PNPs do not show any immunogenic properties and graft rejection [124].

### 4.3. Polymeric Nanostructures

Polymers are substances that are made up of small organic molecules (i.e., monomers) linked together in long, repeating chains. The polymer’s chain length is determined by the molecular weight of separate monomers and the degree of polymerization. Changes in polymer production processes can enhance the functional properties of synthetic polymers, and they can further be modified accordingly to be used for specific applications. The majority of the polymers are biomaterials commonly used in healthcare sectors, specifically in drug delivery systems, surgical tools, tissue engineering, and medical device coatings [125].

Polyethylene, polypropylene, polystyrene, polyvinyl chloride, polylactides, polytetrafluoroethylene, polymethylmethacrylate, polyamides (nylon), polysiloxanes (silicone), and polyurethanes (PUs) are examples of synthetic polymers. In contrast, DNA, hyaluronic acid, gelatine, and collagen are a few examples of natural polymers [126,127]. Polymers have several advantages, including ease of manufacture, inexpensive cost, and the ability to be employed in a variety of dressing materials. Synthetic polymeric nanostructures are mainly used as graft materials in tissue engineering applications and as a biological carrier in drug delivery applications as well as in the preparation of medical devices. Furthermore, they are less expensive than biological scaffolds and have a longer shelf life [77].

In a scientific study conducted on rats, researchers discovered that PU promotes cellular proliferation indirectly by successfully inducing angiogenesis and reepithelization [128]. In addition to PU, polymeric hydrogels were also found to have the potential to aid in tissue repair. The hydrogel scaffold stimulates inflammatory cell infiltration in the early stages of healing and enhances the necessity of angiogenic cells in later stages of recovery, resulting in the development of delayed healing. In the case of any wound, cells and cytokines are added to the polymer, which additionally promotes the development of new blood vessels and the improvement of the milieu around the wound [129]. As a result, these polymeric compositions could be useful in the treatment of both normal and delayed infectious wounds.

According to the recent discovery, cross-linked polymer chains may swell from their native state when present in an aqueous environment. This distinguishing feature of polymers highlights the important function that polymers play in wound healing. For example, gelatin, a natural polymer synthesized from collagen, has mostly been used to make biodegradable and biocompatible wound dressing products. It was further reported that the porosity and interfiber spacing of a gelatin scaffold play a major role in skin repair [130]. Gelatin-based scaffolds also showed improved wound healing in rats [131].

Fibrin is also a different natural polymer derived from fibrinogen and then polymerized into fibrin, and this process is catalyzed by the enzyme thrombin. Fibrin has several unique qualities, including the ability to reduce inflammation, increase immunological response, and improve cell adhesion, and it is commonly employed in tissue engineering and wound healing [132]. Hydrogels have many characteristics, including their mechanical strength, water retaining ability, and biocompatibility that helps in preventing tissue dehydration. Thus, hydrogels can be used to make bandages and dressings for wounds and ulcers. Alternatively, scaffolds deliver strong mechanical support as well as permit growth factor delivery at the targeted sites, both of which are critical properties needed for tissue regeneration. Yet, the main disadvantage of scaffolds is that they must be implanted at the target site, limiting their application [133].

At present, hydrogel usage is more prevalent due to its injectable nature and the ability to attain a defect shape. Additionally, hydrogels are also exceedingly elastic and flexible due to their capacity to hold enormous amounts of water. The delivery of growth factors by hydrogels and scaffolds follows a similar delivery mechanism involving covalent bonding between growth factors and hydrogels. FGF-2 combined with a 3% hydroxypropyl cellulose (HPC) gel for periodontal tissue regeneration yielded minimal results in recent clinical research [134]. Another clinical study indicated that injecting recombinant human FGF-2 combined with a biodegradable gelatine hydrogel into the knee during surgery in osteoarthritic patients was safe and effective [135]. Other clinical experiments using hydrogels include the use of FGF in a fibrin gel to treat cervical spinal cord damage, which exhibited effective nerve regeneration characteristics [136].

### 4.4. Liposomes

Liposomes are comprised of hydrophobic shells and a hydrophilic inner core. They have been widely investigated as carriers for wound healing due to their ability to encapsulate hydrophobic and hydrophilic therapeutic agents, such as antibiotics and bactericidal ions [64]. Liposomes can enable the diffusion of therapeutic agents into bacterial cells and therefore upsurge the drug concentration intracellularly to inhibit bacterial growth and biofilm formation. The liposome composition can be explicitly modified to enhance uptake and sorption via the bacterial cellular membrane. So far, numerous liposomes (with various surface alterations) loaded with antibiotics have been studied for bacterial infections inhibition [64,65].

Liposomes carry a negative charge and bind more efficiently with the bacterial cell membrane carrying the positive charge than with the negative or neutral carrying charge [54,64]. For instance, liposomes (cationic) laden with vancomycin were effective against methicillin-resistant *Staphylococcus aureus* or *S. epidermidis* [65]. Liposome-based carriers are extremely biocompatible and have comparatively low immunogenicity as the conformation of the lipid bilayer can be modified. Furthermore, liposomes can be altered and implanted in wound bandages to deliver therapeutic agents constantly and adequately to inhibit bacteria to the wounded area [66,67].

### 4.5. Lipid NPs

Lipid NPs are propitious vehicles for medicinal agents, for example, drugs [137], growth factors [138], and small interfering RNA (siRNA) [139]. Two kinds of lipid NPs, i.e., solid lipid NPs (SLNs) and nanosized lipid carriers (NLCs), have been formed as efficient carriers for wound treatment [68,69]. These carriers deliver biocompatible components that proficiently save medicinal agents from degradation and offer drug release sustainably. SLNs and NLCs displayed an advantage over the conventional delivery vehicles. Additionally, their small sizes may enable diffusion into biofilm, thus encouraging close contact between drug and bacterial cells.

The antimicrobial peptide LL37 plays a key role in defending the human body against pathogenic infections [70]. Peptide LL37 was delivered by SLNs to the wound and observed to be released continuously for 14 days. In addition, the peptide encapsulation in SLNs prevented them from degradation for a much increased duration, and the continuous LL37 release displayed strong bactericidal properties against *S. aureus* and *E. coli*, which eventually boosted wound healing. NLCs or SLNs have been transformed into sponges [137], hydrogels [68], and films [140] to transport and release active molecules at specific sites and aid wound recovery. For instance, a SLN carrying silver sulfadiazine (SSD) was captured into a chitosan hydrogel matrix for safe SSD delivery to wounds [68]. SSD–SLN enabled the SSD distribution and diffusion into biofilm, leading to protracted and enhanced antibacterial activity. Specifically, in contrast to free SSD, SSD–SLN improved the antibacterial activity toward planktonic bacteria and *P. aeruginosa* biofilm. The repressive action of SSD–SLN on the planktonic growth was extended to 48 h at 18.75 μg/mL minimum concentration [68].

## 5. Limitations of NPs in Wound Healing

NPs have an admirable capability to encourage wound recovery, and there are still tremendous prospects for their implementation and progress in the future. Yet, it ought to be noteworthy that the wound surface is not secured by intact skin. NPs used during wound healing are in direct contact with the wound tissue, and therefore the biological safety of the NPs is crucial before application [141]. The frequently described transdermal noxiousness of NPs is skin irritation and allergies. For instance, carbon nanotubes and nickel NPs have been reported to cause skin hypersensitivity because of the ions released and surface coverings from NPs [142]. Research displayed that NP exposure to transdermal skin can exacerbate skin inflammation, irritation, and psoriasis [143,144]. Some studies also showed oxidative stress, autophagy, and programmed cell death in fibroblasts and keratinocytes when exposed to NPs [145]. The noxiousness of NPs (nickel, gold, and silver, etc.) depends on the shape, size, surface charge, steadiness, and concentration. Thus, when novel NPs are developed for wound treatment, it is crucial to adjust the physicochemical properties to decrease the harmfulness toward skin cells [146]. The increase in the NP stability decreases dermatitis caused by NPs. Stabilizers, for instance, metal shells, a polymer, or surfactants, can be used [147,148].

Furthermore, low sensitization materials should be used for NP surface coating to reduce skin irritation. Additionally, it has been stated that NPs lead to DNA damage and reduce gene methylation, signifying the possibility of cell canceration [149,150]. Yet, there is no clear indication to demonstrate that NPs can lead to malignant alterations and hereditary gene mutations in skin cells. In addition, there is no clear evidence that the prolonged exposure of NPs to percutaneous and deposition in the skin will result in profound effects, which is essential to establish by extended exposure studies in the future. Once NPs enter the body, they directly contact blood cells via damaged blood vessels in lesions and enter the blood circulation, leading to hemolysis. A few metal NPs, such as AgNPs and ZnO NPs, have been shown to cause hemolysis. To overcome the aforementioned complication, the material’s physicochemical properties can be adjusted, or the surface of NPs can be wrapped with biologically active substances, for example, polysaccharides and phospholipids [151].

Another problem is that NPs will spread all over the body and to various organs, instigating multisystem defects. Compared with the initial concentration of the NPs, the NP amount after entering the blood circulation decreases significantly and might be partially defecated in urine and feces. In animal studies, weight loss and death have been detected. However, there is no convincing indication concerning whether NPs will lead to organ damage and/or tumors in practical use. In addition, the introduction during pregnancy will disturb progeny [152]. Generally, NP toxicity evaluation in wound curing have primarily focused on local acute adverse responses. In contrast, several studies have been based on metal NPs, carbonaceous NPs, and nanotubes, while nanofibers, nanofilms, and other innovative NPs are still infrequently considered [153]. Thus, an immediate NP toxicity investigation is required to resolve these complications.

## 6. Future Perspectives and Challenges

### 6.1. Lacunae in the Current Research

Here, NPs have shown a beneficial influence during each wound healing process. However, the primary limiting factor for NP implications in wound healing is the formulation costing. The formulations might be minimized by reducing NP doses. Primarily, the addition of adjuvants with a lower price or formulating composite NPs can boost the therapeutic efficiency [154]. Second, specific or sustained drug delivery and release can be exploited, such as microneedle methods, NP layered self-assembly, and controlled release of laden medicines maintained by nanoscale systems [155,156,157]. Improving formulations and procedures is also an efficient approach to lower production expenses. Most recent investigations have been studied in vitro, and in vivo research needs to be upgraded.

Rats, mice, rabbits, and pigs were investigated for the research. Due to the alterations in size, price, and availability of the animals, the most commonly used ones were mice and rats. However, their skin geomorphologies and wound healing processes are dissimilar to that of humans. In contrast, pig’s skin is the utmost analogous to humans. Pigs are not extensively used in wound healing investigations due to the high price and bulky nature of experiments with bigger animals [158]. The processes and drugs for the treatment of wounds are changing quickly, and NPs are being used on wounds in numerous formulations. Nevertheless, the mechanism via which the NPs help in wound recovery has been investigated superficially. For instance, the frequently described pathways for proliferation and inflammation are transforming growth factor-β1/ Suppressor of Mothers against Decapentaplegic (TGF-β1/SMAD) signaling pathway and macrophage polarization, respectively. The mechanism by which NPs aid in wound healing after reconstruction has rarely been examined. There are various categories of NPs with diverse characteristics, and wound healing advancements should also be multidimensional. In the future, more profound and advanced mechanisms need to be discovered, which would expand treatment approaches and evade redundant side effects [159,160].

### 6.2. Future Challenges

Since NPs have an admirable drug carrying capacity, a large number of novel drugs have been laden onto NPs. For immune skin infections, the development of various biological agents has progressively substituted traditional drugs. The loading of monoclonal antibodies onto NPs also increases the absorption rate and efficiency of the drug. Even though the present study has established that NPs may play a beneficial role in all stages of wound recovery, the same NPs cannot be effective during the whole course, and diverse kinds of NPs may be needed at various phases. It is difficult to visually know each phase’s dividing point; thus, developing real-time monitoring of the wound state is crucial. Recently, ZnO nanowire-based self-powered implantable electronic skin altered by enzymes (uricase and urease) has been produced for human health detection transdermally, including humidity, temperature, blood pressure, electrolyte metabolites, etc. [161,162]. In the future, with the help of this technology, electronic skin monitoring of the inflammatory factors, pH, humidity, and signaling pathway proteins can be developed. This will help the medical practitioner regulate wound treatment precisely and select suitable NPs based on the real-time situation. The wound treatment follows the filling of imperfections and needs complete functional and visual recovery. Currently, researchers have efficaciously applied NPs in wound healing and averted scar development. Further, nanotechnology has a great possibility in hair follicle regeneration, paresthesia regulation, and abnormal pigmentation improvement. Recently, the development of electronic skin and the combination of electronic technology and nanotechnology have provided new-found concepts for paresthesia recovery after wound development and brought a smart idea for wound healing.

## 7. Conclusions

Nowadays, there is a rapid increase in the application of nanomaterials for wound treatment. The present work has reviewed the recent advancements in nanomaterials facilitating wound healing and the mechanisms involved. Most of the literature is on the promotion of hemostasis, anti-infection, immunoregulation, and proliferation; nonetheless, there is research lacuna for the proper mechanisms and postwound modifications. Owing to the peculiar physicochemical and biological properties of NPs, possessing a highly specific surface area also showed potential application in the wound dressings for the delivery and release of the therapeutic agents in a sustainable way. In addition, NPs for wound healing can absorb light and further transform it to heat or ROS, ultimately resulting in the bacterial death present in the wounds. Further, NPs may be unified for developing a keen wound dressing to treat microbial infections based on endogenous triggers, such as pH, temperature, enzymes, and toxins secreted by the bacteria.

In spite of the vast growth in the efficient NP-based wound dressings for bacteria detection and treatment, there are yet numerous unavoidable hurdles, for example, reproducibility, stability, toxicity, and histocompatibility that vastly hinder the translation of NPs from a laboratory experiment to the clinical application. In addition, understanding the behavior of NP-based wound dressings in vivo is usually examined in animal trials. Hence, it is vital to find an alternative solution for the preclinical studies because of the varied alterations between the human and animal models. Recently, there has been an increase in the interest and breakthrough for intelligent wound dressings in relation to the NPs that is able to detect bacterial infections within the time period and treat bacteria without removing the dressings from the wound. This quickly monitors and treats bacterial infections via the multimodal method for synergistic and effective therapy. Consequently, NP-based wound healing holds future hope for the detection and therapy of infections in wounds.

## Figures and Tables

**Table 1:** Function of various biomolecules involved in wound healing.

Growth Factor	Cell Sources	Role	Reference
Platelet-derived growth factor (PDGF)			
	Platelets, macrophages, epidermal cells, keratinocytes	Neutrophils and fibroblasts migration; triggers macrophages	[17,18]
Transforming growth factor (TGF)-β family			
	Platelets, macrophages	Chemoattractant for inflammatory cells; clot formation; fibrosis	[18,19]
Vascular endothelial growth factor (VEGF)			
	Platelets, macrophages, fibroblasts, epidermal cells	Angiogenesis and migration of endothelial cells	[18,20]
Endothelial growth factor (EGF) family (TGF-α and EGF)			
	Platelets, fibroblasts,	Mesenchymal; migration of keratinocytes, fibroblast and endothelial cells	[20,21]
Insulin-like growth factor (IGF) family			
	Plasma, platelets	Stimulate extracellular matrix deposition and fibroblast growth; protein and DNA synthesis	[22,23]
Fibroblast growth factor (FGF) family (FGF and keratinocyte growth factor (KGF))			
	Fibroblasts, Endothelial cells, keratinocytes	Cell proliferation; cell stemness; dedifferentiation; inflammation; angiogenesis	[22]
Interleukin			
	Macrophages, keratinocytes, endothelial cells, and neutrophils	Release of proinflammatory cytokines; differentiation, activation, and proliferation of leukocytes, endothelial cells, keratinocytes, and fibroblasts	[15,24]
Tumor necrosis factor (TNF)-α			
	Neutrophils, macrophages	Promotes the formation of the extracellular matrix; release of inflammatory cytokines	[25]

## Data Availability

This study did not report any data.

## References

[1] Dąbrowska A., Spano F., Derler S., Adlhart C., Spencer N., Rossi R. (2018). The relationship between skin function, barrier properties, and body-dependent factors. Ski. Res. Technol..

[2] Hoversten K.P., Kiemele L.J., Stolp A.M., Takahashi P.Y., Verdoorn B.P. (2020). Prevention, Diagnosis, and Management of Chronic Wounds in Older Adults. Mayo Clin. Proc..

[3] Spampinato S.F., Caruso G.I., De Pasquale R., Sortino M.A., Merlo S. (2020). The Treatment of Impaired Wound Healing in Diabetes: Looking among Old Drugs. Pharmaceuticals.

[4] Wang M., Huang X., Zheng H., Tang Y., Zeng K., Shao L., Li L. (2021). Nanomaterials applied in wound healing: Mechanisms, limitations and perspectives. J. Control. Release.

[5] Berthet M., Gauthier Y., Lacroix C., Verrier B., Monge C. (2017). Nanoparticle-Based Dressing: The Future of Wound Treatment?. Trends Biotechnol..

[6] Mihai M.M., Dima M.B., Dima B., Holban A.M. (2019). Nanomaterials for Wound Healing and Infection Control. Materials.

[7] Han G., Ceilley R. (2017). Chronic Wound Healing: A Review of Current Management and Treatments. Adv. Ther..

[8] Homaeigohar S., Boccaccini A.R. (2020). Antibacterial biohybrid nanofibers for wound dressings. Acta Biomater..

[9] Goswami L., Kushwaha A., Singh A., Saha P., Choi Y., Maharana M., Patil S.V., Kim B.S. (2022). Nano-Biochar as a Sustainable Catalyst for Anaerobic Digestion: A Synergetic Closed-Loop Approach. Catalysts.

[10] Victor P., Sarada D., Ramkumar K.M. (2020). Pharmacological activation of Nrf2 promotes wound healing. Eur. J. Pharmacol..

[11] Matter M.T., Probst S., Läuchli S., Herrmann I.K. (2020). Uniting Drug and Delivery: Metal Oxide Hybrid Nanotherapeutics for Skin Wound Care. Pharmaceutics.

[12] Rodrigues M., Kosaric N., Bonham C.A., Gurtner G.C. (2019). Wound Healing: A Cellular Perspective. Physiol. Rev..

[13] Periayah M.H., Halim A.S., Mat Saad A.Z. (2017). Mechanism Action of Platelets and Crucial Blood Coagulation Pathways in Hemostasis. Int. J. Hematol. Oncol. Stem Cell Res..

[14] Pradhan S., Khatlani T., Nairn A.C., Vijayan K.V. (2017). The heterotrimeric G protein Gβ1 interacts with the catalytic subunit of protein phosphatase 1 and modulates G protein–coupled receptor signaling in platelets. J. Biol. Chem..

[15] Sharifi S., Hajipour M.J., Gould L., Mahmoudi M. (2021). Nanomedicine in Healing Chronic Wounds: Opportunities and Challenges. Mol. Pharm..

[16] Rumbaut R.E., Thiagarajan P. (2010). Platelet-Vessel Wall Interactions in Hemostasis and Thrombosis. Colloq. Ser. Integr. Syst. Physiol. Mol. Funct..

[17] Yamakawa S., Hayashida K. (2019). Advances in surgical applications of growth factors for wound healing. Burn. Trauma.

[18] Branski L.K., Pereira C.T., Herndon D.N., Jeschke M.G. (2006). Gene therapy in wound healing: Present status and future directions. Gene Ther..

[19] Pakyari M., Farrokhi A., Maharlooei M.K., Ghahary A. (2013). Critical Role of Transforming Growth Factor Beta in Different Phases of Wound Healing. Adv. Wound Care.

[20] Bao P., Kodra A., Tomic-Canic M., Golinko M.S., Ehrlich H.P., Brem H. (2009). The Role of Vascular Endothelial Growth Factor in Wound Healing. J. Surg. Res..

[21] Bodnar R.J. (2013). Epidermal Growth Factor and Epidermal Growth Factor Receptor: The Yin and Yang in the Treatment of Cutaneous Wounds and Cancer. Adv. Wound Care.

[22] Barrientos S., Stojadinovic O., Golinko M.S., Brem H., Tomic-Canic M. (2008). PERSPECTIVE ARTICLE: Growth factors and cytokines in wound healing. Wound Repair Regen..

[23] Achar R.A.N., Silva T.C., Achar E., Martines R.B., Machado J.L.M. (2014). Use of insulin-like growth factor in the healing of open wounds in diabetic and non-diabetic rats. Acta Cir. Bras..

[24] Feliciani C., Gupta A., Saucier D. (1996). Keratinocytes and Cytokine/Growth Factors. Crit. Rev. Oral Biol. Med..

[25] Ritsu M., Kanno E., Tanno H., Imai Y., Maruyama R., Tachi M., Kawakami K., Ishii K. (2017). Critical role of tumor necrosis factor-α in the early process of wound healing in skin. J. Dermatol. Dermatol. Surg..

[26] Cañedo-Dorantes L., Cañedo-Ayala M. (2019). Skin Acute Wound Healing: A Comprehensive Review. Int. J. Inflamm..

[27] Young A., McNaught C.-E. (2011). The physiology of wound healing. Surgery.

[28] Kratofil R.M., Kubes P., Deniset J.F. (2017). Monocyte Conversion During Inflammation and Injury. Arter. Thromb. Vasc. Biol..

[29] Velnar T., Bailey T., Smrkolj V. (2009). The Wound Healing Process: An Overview of the Cellular and Molecular Mechanisms. J. Int. Med. Res..

[30] Landén N.X., Li D., Ståhle M. (2016). Transition from inflammation to proliferation: A critical step during wound healing. Cell. Mol. Life Sci..

[31] Fathke C., Wilson L., Hutter J., Kapoor V., Smith A., Hocking A., Isik F. (2004). Contribution of Bone Marrow–Derived Cells to Skin: Collagen Deposition and Wound Repair. Stem Cells.

[32] Kiwanuka E., Junker J., Eriksson E. (2012). Harnessing Growth Factors to Influence Wound Healing. Clin. Plast. Surg..

[33] Xue M., Jackson C.J. (2015). Extracellular Matrix Reorganization During Wound Healing and Its Impact on Abnormal Scarring. Adv. Wound Care.

[34] Guo S., DiPietro L.A. (2010). Factors Affecting Wound Healing. J. Dent. Res..

[35] Ayuk S.M., Abrahamse H., Houreld N.N. (2016). The Role of Matrix Metalloproteinases in Diabetic Wound Healing in relation to Photobiomodulation. J. Diabetes Res..

[36] Brem H., Stojadinovic O., Diegelmann R.F., Entero H., Lee B., Pastar I., Golinko M.S., Rosenberg H., Tomic-Canic M. (2007). Molecular Markers in Patients with Chronic Wounds to Guide Surgical Debridement. Mol. Med..

[37] Clinton A., Carter T. (2015). Chronic Wound Biofilms: Pathogenesis and Potential Therapies. Lab. Med..

[38] Piipponen M., Li D., Landén N.X. (2020). The Immune Functions of Keratinocytes in Skin Wound Healing. Int. J. Mol. Sci..

[39] Bruce E.D., Christie M.S. (2016). Toxicological outcomes and pharmacological needs in chronic wound healing. EC Pharmacol. Toxicol..

[40] Frykberg R.G., Banks J. (2015). Challenges in the Treatment of Chronic Wounds. Adv. Wound Care.

[41] Sinno H., Prakash S. (2013). Complements and the Wound Healing Cascade: An Updated Review. Plast. Surg. Int..

[42] McDaniel J.C., Roy S., Wilgus T.A. (2013). Neutrophil activity in chronic venous leg ulcers—A target for therapy?. Wound Repair Regen..

[43] Qiao Y., He J., Chen W., Yu Y., Li W., Du Z., Xie T., Ye Y., Hua S.Y., Zhong D. (2020). Light-Activatable Synergistic Therapy of Drug-Resistant Bacteria-Infected Cutaneous Chronic Wounds and Nonhealing Keratitis by Cupriferous Hollow Nanoshells. ACS Nano.

[44] Chen C., Liu Y., Sun L., Chen G., Wu X., Ren J., Zhao Y. (2019). Antibacterial porous microcarriers with a pathological state responsive switch for wound healing. ACS Appl. Bio Mater..

[45] Kumar S., Majhi R.K., Singh A., Mishra M., Tiwari A., Chawla S., Guha P., Satpati B., Mohapatra H., Goswami L. (2019). Carbohydrate-Coated Gold–Silver Nanoparticles for Efficient Elimination of Multidrug Resistant Bacteria and In Vivo Wound Healing. ACS Appl. Mater. Interfaces.

[46] Shin J.U., Gwon J., Lee S.-Y., Yoo H.S. (2018). Silver-Incorporated Nanocellulose Fibers for Antibacterial Hydrogels. ACS Omega.

[47] Hu C., Zhang F., Kong Q., Lu Y., Zhang B., Wu C., Luo R., Wang Y. (2019). Synergistic Chemical and Photodynamic Antimicrobial Therapy for Enhanced Wound Healing Mediated by Multifunctional Light-Responsive Nanoparticles. Biomacromolecules.

[48] Jiang S., Ma B.C., Huang W., Kaltbeitzel A., Kizisavas G., Crespy D., Zhang K.A.I., Landfester K. (2018). Visible light active nanofibrous membrane for antibacterial wound dressing. Nanoscale Horiz..

[49] Das M., Goswami U., Kandimalla R., Kalita S., Ghosh S.S., Chattopadhyay A. (2019). Iron–Copper Bimetallic Nanocomposite Reinforced Dressing Materials for Infection Control and Healing of Diabetic Wound. ACS Appl. Biomater..

[50] Yan X., Fang W.-W., Xue J., Sun T.-C., Dong L., Zha Z., Qian H., Song Y.-H., Zhang M., Gong X. (2019). Thermoresponsive in Situ Forming Hydrogel with Sol–Gel Irreversibility for Effective Methicillin-Resistant *Staphylococcus aureus* Infected Wound Healing. ACS Nano.

[51] Wang Y., Lu Y., Zhang J., Hu X., Yang Z., Guo Y., Wang Y. (2019). A synergistic antibacterial effect between terbium ions and reduced graphene oxide in a poly(vinyl alcohol)–alginate hydrogel for treating infected chronic wounds. J. Mater. Chem. B.

[52] Zhu M., Liu P., Shi H., Tian Y., Ju X., Jiang S., Li Z., Wu M., Niu Z. (2018). Balancing antimicrobial activity with biological safety: Bifunctional chitosan derivative for the repair of wounds with Gram-positive bacterial infections. J. Mater. Chem. B.

[53] de Lima G.G., de Lima D.W., de Oliveira M.J., Lugão A.B., Alcântara M.T., Devine D.M., de Sá M.J. (2018). Synthesis and in vivo behavior of PVP/CMC/agar hydrogel membranes impregnated with silver nanoparticles for wound healing applications. ACS Appl. Bio Mater..

[54] Wang S., Yan C., Zhang X., Shi D., Chi L., Luo G., Deng J. (2018). Antimicrobial peptide modification enhances the gene delivery and bactericidal efficiency of gold nanoparticles for accelerating diabetic wound healing. Biomater. Sci..

[55] Tong C., Zou W., Ning W., Fan J., Li L., Liu B., Liu X. (2018). Synthesis of DNA-guided silver nanoparticles on a graphene oxide surface: Enhancing the antibacterial effect and the wound healing activity. RSC Adv..

[56] Jin C., Liu X., Tan L., Cui Z., Yang X., Zheng Y., Wu S. (2018). Ag/AgBr-loaded mesoporous silica for rapid sterilization and promotion of wound healing. Biomater. Sci..

[57] Yuwen L., Sun Y., Tan G., Xiu W., Zhang Y., Weng L., Teng Z., Wang L. (2018). MoS_2_@polydopamine-Ag nanosheets with enhanced antibacterial activity for effective treatment of Staphylococcus aureus biofilms and wound infection. Nanoscale.

[58] George L., Bavya M., Rohan K.V., Srivastava R. (2017). A therapeutic polyelectrolyte–vitamin C nanoparticulate system in polyvinyl alcohol–alginate hydrogel: An approach to treat skin and soft tissue infections caused by *Staphylococcus aureus*. Colloids Surf. B Biointerfaces.

[59] Ehterami A., Salehi M., Farzamfar S., Vaez A., Samadian H., Sahrapeyma H., Mirzaii M., Ghorbani S., Goodarzi A. (2018). In vitro and in vivo study of PCL/COLL wound dressing loaded with insulin-chitosan nanoparticles on cutaneous wound healing in rats model. Int. J. Biol. Macromol..

[60] Hasan N., Cao J., Lee J., Hlaing S.P., Oshi M.A., Naeem M., Ki M.-H., Lee B.L., Jung Y., Yoo J.-W. (2019). Bacteria-Targeted Clindamycin Loaded Polymeric Nanoparticles: Effect of Surface Charge on Nanoparticle Adhesion to MRSA, Antibacterial Activity, and Wound Healing. Pharmaceutics.

[61] Aly U.F., Aboutaleb H.A., Abdellatif A.A., Tolba N.S. (2019). Formulation and evaluation of simvastatin polymeric nanoparticles loaded in hydrogel for optimum wound healing purpose. Drug Des. Dev. Ther..

[62] Koudehi M.F., Zibaseresht R. (2019). Synthesis of molecularly imprinted polymer nanoparticles containing gentamicin drug as wound dressing based polyvinyl alcohol/gelatin nanofiber. Mater. Technol..

[63] Hasan N., Cao J., Lee J., Naeem M., Hlaing S.P., Kim J., Jung Y., Lee B.-L., Yoo J.-W. (2019). PEI/NONOates-doped PLGA nanoparticles for eradicating methicillin-resistant *Staphylococcus aureus* biofilm in diabetic wounds via binding to the biofilm matrix. Mater. Sci. Eng. C.

[64] Scriboni A.B., Couto V.M., Ribeiro L.N.D.M., Freires I.A., Groppo F.C., De Paula E., Franz-Montan M., Cogo-Müller K. (2019). Fusogenic Liposomes Increase the Antimicrobial Activity of Vancomycin Against *Staphylococcus aureus* Biofilm. Front. Pharmacol..

[65] Rukavina Z., Šegvić K.M., Filipović-Grčić J., Lovrić J., Vanić Ž. (2018). Azithromycin-loaded liposomes for enhanced topical treatment of methicillin-resistant *Staphyloccocus aureus* (MRSA) infections. Int. J. Pharm..

[66] Monteiro N., Martins M., Martins A., Fonseca N.A., Moreira J.N., Reis R.L., Neves N.M. (2015). Antibacterial activity of chitosan nanofiber meshes with liposomes immobilized releasing gentamicin. Acta Biomater..

[67] Thapa R.K., Kiick K.L., Sullivan M.O. (2020). Encapsulation of collagen mimetic peptide-tethered vancomycin liposomes in collagen-based scaffolds for infection control in wounds. Acta Biomater..

[68] Patel K.K., Surekha D.B., Tripathi M., Anjum M.M., Muthu M.S., Tilak R., Agrawal A.K., Singh S. (2019). Antibiofilm Potential of Silver Sulfadiazine-Loaded Nanoparticle Formulations: A Study on the Effect of DNase-I on Microbial Biofilm and Wound Healing Activity. Mol. Pharm..

[69] Saporito F., Sandri G., Bonferoni M.C., Rossi S., Boselli C., Cornaglia A.I., Mannucci B., Grisoli P., Vigani B., Ferrari F. (2018). Essential oil-loaded lipid nanoparticles for wound healing. Int. J. Nanomed..

[70] Fumakia M., Ho E.A. (2016). Nanoparticles Encapsulated with LL37 and Serpin A1 Promotes Wound Healing and Synergistically Enhances Antibacterial Activity. Mol. Pharm..

[71] Mirzahosseinipour M., Khorsandi K., Hosseinzadeh R., Ghazaeian M., Shahidi F.K. (2020). Antimicrobial photodynamic and wound healing activity of curcumin encapsulated in silica nanoparticles. Photodiagn. Photodyn. Ther..

[72] Zhang Y., Chang M., Bao F., Xing M., Wang E., Xu Q., Huan Z., Guo F., Chang J. (2019). Multifunctional Zn doped hollow mesoporous silica/polycaprolactone electrospun membranes with enhanced hair follicle regeneration and antibacterial activity for wound healing. Nanoscale.

[73] Alvarez G.S., Hélary C., Mebert A.M., Wang X., Coradin T., Desimone M.F. (2014). Antibiotic-loaded silica nanoparticle–collagen composite hydrogels with prolonged antimicrobial activity for wound infection prevention. J. Mater. Chem. B.

[74] Casciaro B., Moros M., Rivera-Fernández S., Bellelli A., de la Fuente J.M., Mangoni M.L. (2017). Gold-nanoparticles coated with the antimicrobial peptide esculentin-1a(1-21)NH_2_ as a reliable strategy for antipseudomonal drugs. Acta Biomater..

[75] Shah M.R., Ali S., Ateeq M., Perveen S., Ahmed S., Bertino M.F., Ali M. (2014). Morphological analysis of the antimicrobial action of silver and gold nanoparticles stabilized with ceftriaxone on *Escherichia coli* using atomic force microscopy. New J. Chem..

[76] Xu C., Akakuru O.U., Ma X., Zheng J., Zheng J., Wu A. (2020). Nanoparticle-Based Wound Dressing: Recent Progress in the Detection and Therapy of Bacterial Infections. Bioconjug. Chem..

[77] Rajendran N.K., Kumar S.S.D., Houreld N.N., Abrahamse H. (2018). A review on nanoparticle based treatment for wound healing. J. Drug Deliv. Sci. Technol..

[78] Hasanin M., Swielam E.M., Atwa N.A., Agwa M.M. (2021). Novel design of bandages using cotton pads, doped with chitosan, glycogen and ZnO nanoparticles, having enhanced antimicrobial and wounds healing effects. Int. J. Biol. Macromol..

[79] Rowe S.E., Wagner N.J., Li L., Beam J.E., Wilkinson A.D., Radlinski L.C., Zhang Q., Miao E.A., Conlon B.P. (2019). Reactive oxygen species induce antibiotic tolerance during systemic Staphylococcus aureus infection. Nat. Microbiol..

[80] Nethi S.K., Das S., Patra C.R., Mukherjee S. (2019). Recent advances in inorganic nanomaterials for wound-healing applications. Biomater. Sci..

[81] Li Y., Tian Y., Zheng W., Feng Y., Huang R., Shao J., Tang R., Wang P., Jia Y., Zhang J. (2017). Composites of Bacterial Cellulose and Small Molecule-Decorated Gold Nanoparticles for Treating Gram-Negative Bacteria-Infected Wounds. Small.

[82] Panáček A., Kvítek L., Smékalová M., Večeřová R., Kolář M., Röderová M., Dyčka F., Šebela M., Prucek R., Tomanec O. (2018). Bacterial resistance to silver nanoparticles and how to overcome it. Nat. Nanotechnol..

[83] Zhang X.-F., Shen W., Gurunathan S. (2016). Silver Nanoparticle-Mediated Cellular Responses in Various Cell Lines: An in Vitro Model. Int. J. Mol. Sci..

[84] Marcato P.D., De Paula L.B., Melo P.S., Ferreira I.R., Almeida A.B.A., Torsoni A., Alves O.L. (2015). In Vivo Evaluation of Complex Biogenic Silver Nanoparticle and Enoxaparin in Wound Healing. J. Nanomater..

[85] Sarhan W.A., Azzazy H.M.E., El-Sherbiny I.M. (2016). Honey/Chitosan Nanofiber Wound Dressing Enriched with *Allium sativum* and *Cleome droserifolia*: Enhanced Antimicrobial and Wound Healing Activity. ACS Appl. Mater. Interfaces.

[86] Ye H., Cheng J., Yu K. (2019). In situ reduction of silver nanoparticles by gelatin to obtain porous silver nanoparticle/chitosan composites with enhanced antimicrobial and wound-healing activity. Int. J. Biol. Macromol..

[87] Kong F., Fan C., Yang Y., Lee B.H., Wei K. (2019). 5-hydroxymethylfurfural-embedded poly (vinyl alcohol)/sodium alginate hybrid hydrogels accelerate wound healing. Int. J. Biol. Macromol..

[88] Thanh N.T., Hieu M.H., Phuong N.T.M., Thuan T.D.B., Thu H.N.T., Thai V.-P., Minh T.D., Dai H.N., Vo V.T., Thi H.N. (2018). Optimization and characterization of electrospun polycaprolactone coated with gelatin-silver nanoparticles for wound healing application. Mater. Sci. Eng. C.

[89] You C., Liping Z., Wang X., Wu P., Ho J.K., Jin R., Zhang L., Shao H., Han C. (2017). Silver nanoparticle loaded collagen/chitosan scaffolds promote wound healing via regulating fibroblast migration and macrophage activation. Sci. Rep..

[90] Liu Y., Shi L., Su L., Van Der Mei H.C., Jutte P.C., Ren Y., Busscher H.J. (2019). Nanotechnology-based antimicrobials and delivery systems for biofilm-infection control. Chem. Soc. Rev..

[91] Le Ouay B., Stellacci F. (2015). Antibacterial activity of silver nanoparticles: A surface science insight. Nano Today.

[92] Chatterjee A.K., Chakraborty R., Basu T. (2014). Mechanism of antibacterial activity of copper nanoparticles. Nanotechnology.

[93] Alizadeh S., Seyedalipour B., Shafieyan S., Kheime A., Mohammadi P., Aghdami N. (2019). Copper nanoparticles promote rapid wound healing in acute full thickness defect via acceleration of skin cell migration, proliferation, and neovascularization. Biochem. Biophys. Res. Commun..

[94] Deryabin D.G., Aleshina E.S., Vasilchenko A.S., Deryabina T.D., Efremova L.V., Karimov I.F., Korolevskaya L.B. (2013). Investigation of copper nanoparticles antibacterial mechanisms tested by luminescent *Escherichia coli* strains. Nanotechnol. Russ..

[95] Grass G., Rensing C., Solioz M. (2011). Metallic copper as an antimicrobial surface. Appl. Environ. Microbiol..

[96] Kornblatt A.P., Nicoletti V.G., Travaglia A. (2016). The neglected role of copper ions in wound healing. J. Inorg. Biochem..

[97] LewisOscar F., MubarakAli D., Nithya C., Priyanka R., Gopinath V., Alharbi N.S., Thajuddin N. (2015). One pot synthesis and anti-biofilm potential of copper nanoparticles (CuNPs) against clinical strains of *Pseudomonas aeruginosa*. Biofouling.

[98] Cady N.C., Behnke J.L., Strickland A.D. (2011). Copper-Based Nanostructured Coatings on Natural Cellulose: Nanocomposites Exhibiting Rapid and Efficient Inhibition of a Multi-Drug Resistant Wound Pathogen, *A. baumannii*, and Mammalian Cell Biocompatibility In Vitro. Adv. Funct. Mater..

[99] Li Q., Lu F., Zhou G., Yu K., Lu B., Xiao Y., Dai F., Wu D., Lan G. (2017). Silver Inlaid with Gold Nanoparticle/Chitosan Wound Dressing Enhances Antibacterial Activity and Porosity, and Promotes Wound Healing. Biomacromolecules.

[100] Arafa M.G., El-Kased R.F., Elmazar M.M. (2018). Thermoresponsive gels containing gold nanoparticles as smart antibacterial and wound healing agents. Sci. Rep..

[101] Akturk O., Kismet K., Yasti A.C., Kuru S., E Duymus M., Kaya F., Caydere M., Hucumenoglu S., Keskin D. (2016). Collagen/gold nanoparticle nanocomposites: A potential skin wound healing biomaterial. J. Biomater. Appl..

[102] Gu H., Ho P.L., Tong E., Wang L., Xu B. (2003). Presenting Vancomycin on Nanoparticles to Enhance Antimicrobial Activities. Nano Lett..

[103] Norman S., Stone J.W., Gole A., Murphy C., Sabo-Attwood T.L. (2008). Targeted Photothermal Lysis of the Pathogenic Bacteria, *Pseudomonas aeruginosa*, with Gold Nanorods. Nano Lett..

[104] Gil-Tomás J., Tubby S., Parkin I.P., Narband N., Dekker L., Nair S.P., Wilson M., Street C. (2007). Lethal photosensitisation of *Staphylococcus aureus* using a toluidine blue O–tiopronin–gold nanoparticle conjugate. J. Mater. Chem..

[105] Sherwani M.A., Tufail S., Khan A.A., Owais M. (2015). Gold Nanoparticle-Photosensitizer Conjugate Based Photodynamic Inactivation of Biofilm Producing Cells: Potential for Treatment of *C. albicans* Infection in BALB/c Mice. PLoS ONE.

[106] Naraginti S., Kumari P.L., Das R.K., Sivakumar A., Patil S.H., Andhalkar V.V. (2016). Amelioration of excision wounds by topical application of green synthesized, formulated silver and gold nanoparticles in albino Wistar rats. Mater. Sci. Eng. C.

[107] Volkova N., Yukhta M., Pavlovich O., Goltsev A. (2016). Application of Cryopreserved Fibroblast Culture with Au Nanoparticles to Treat Burns. Nanoscale Res. Lett..

[108] Hsu S.-H., Chang Y.-B., Tsai C.-L., Fu K.-Y., Wang S.-H., Tseng H.-J. (2011). Characterization and biocompatibility of chitosan nanocomposites. Colloid. Sur. B.

[109] Raghupathi K.R., Koodali R.T., Manna A.C. (2011). Size-Dependent Bacterial Growth Inhibition and Mechanism of Antibacterial Activity of Zinc Oxide Nanoparticles. Langmuir.

[110] Pati R., Mehta R.K., Mohanty S., Padhi A., Sengupta M., Vaseeharan B., Goswami C., Sonawane A. (2014). Topical application of zinc oxide nanoparticles reduces bacterial skin infection in mice and exhibits antibacterial activity by inducing oxidative stress response and cell membrane disintegration in macrophages. Nanomed. Nanotechnol. Biol. Med..

[111] Shahzadi L., Chaudhry A.A., Aleem A.R., Malik M.H., Ijaz K., Akhtar H., Alvi F., Khan A.F., Rehman I.U., Yar M. (2018). Development of K-doped ZnO nanoparticles encapsulated crosslinked chitosan based new membranes to stimulate angiogenesis in tissue engineered skin grafts. Int. J. Biol. Macromol..

[112] Balaure P.C., Holban A.M., Grumezescu A.M., Mogoşanu G.D., Bălşeanu T.A., Stan M.S., Mogoantă L. (2019). In vitro and in vivo studies of novel fabricated bioactive dressings based on collagen and zinc oxide 3D scaffolds. Int. J. Pharm..

[113] Rakhshaei R., Namazi H. (2017). A potential bioactive wound dressing based on carboxymethyl cellulose/ZnO impregnated MCM-41 nanocomposite hydrogel. Mater. Sci. Eng. C.

[114] Yang G., Zhang M., Qi B., Zhu Z., Yao J., Yuan X., Sun D. (2018). Nanoparticle-Based Strategies and Approaches for the Treatment of Chronic Wounds. J. Biomater. Tissue Eng..

[115] Wong I.Y., Bhatia S.N., Toner M. (2013). Nanotechnology: Emerging tools for biology and medicine. Genes Dev..

[116] Zhu J., Marchant R.E. (2011). Design properties of hydrogel tissue-engineering scaffolds. Expert Rev. Med. Devices.

[117] Giano M.C., Pochan D.J., Schneider J.P. (2011). Controlled biodegradation of Self-assembling β-hairpin Peptide hydrogels by proteolysis with matrix metalloproteinase-13. Biomaterials.

[118] Haines-Butterick L., Rajagopal K., Branco M., Salick D., Rughani R., Pilarz M., Lamm M.S., Pochan D.J., Schneider J. (2007). Controlling hydrogelation kinetics by peptide design for three-dimensional encapsulation and injectable delivery of cells. Proc. Natl. Acad. Sci. USA.

[119] Wang S., Nagrath D., Chen P.C., Berthiaume F., Yarmush M.L. (2008). Three-dimensional primary hepatocyte culture in synthetic self-assembling peptide hydrogel. Tissue Eng. Part A.

[120] Webber M.J., Tongers J., Renault M.-A., Roncalli J.G., Losordo D.W., Stupp S.I. (2010). Development of bioactive peptide amphiphiles for therapeutic cell delivery. Acta Biomater..

[121] Capito R.M., Azevedo H.S., Velichko Y.S., Mata A., Stupp S.I. (2008). Self-Assembly of Large and Small Molecules into Hierarchically Ordered Sacs and Membranes. Science.

[122] Jayawarna V., Smith A., Gough J.E., Ulijn R.V. (2007). Three-dimensional cell culture of chondrocytes on modified di-phenylalanine scaffolds. Biochem. Soc. Trans..

[123] Smith A.M., Williams R.J., Tang C., Coppo P., Collins R.F., Turner M.L., Ulijn R.V. (2008). Fmoc-diphenylalanine self assembles to a hydrogel via a novel architecture based on π–π interlocked β-sheets. Adv. Mater..

[124] Mohamed A., Xing M. (2012). Nanomaterials and nanotechnology for skin tissue engineering. Int. J. Burn. Trauma.

[125] Bhat S., Kumar A. (2013). Biomaterials and bioengineering tomorrow’s healthcare. Biomatter.

[126] Ramasamy M., Lee J. (2016). Recent Nanotechnology Approaches for Prevention and Treatment of Biofilm-Associated Infections on Medical Devices. BioMed Res. Int..

[127] Ngo Y.H., Li D., Simon G., Garnier G. (2011). Paper surfaces functionalized by nanoparticles. Adv. Colloid Interface Sci..

[128] Heit Y.I., Dastouri P., Helm D.L., Pietramaggiori G., Younan G., Erba P., Münster S., Orgill D.P., Scherer S.S. (2012). Foam Pore Size Is a Critical Interface Parameter of Suction-Based Wound Healing Devices. Plast. Reconstr. Surg..

[129] Jiang B., Larson J.C., Drapala P.W., Pérez-Luna V.H., Kang-Mieler J.J., Brey E.M. (2012). Investigation of lysine acrylate containing poly(N-isopropylacrylamide) hydrogels as wound dressings in normal and infected wounds. J. Biomed. Mater. Res. Part B Appl. Biomater..

[130] Powell H.M., Boyce S.T. (2008). Fiber density of electrospun gelatin scaffolds regulates morphogenesis of dermal–epidermal skin substitutes. J. Biomed. Mater. Res. Part A.

[131] Bilgic H., Demiriz M., Ozler M., Ide T., Dogan N., Gumus S., Kiziltay A., Endogan T., Hasirci N. (2013). Gelatin Based Scaffolds and Effect of EGF Dose on Wound Healing. J. Biomater. Tissue Eng..

[132] Patel H., Bonde M., Srinivasan G. (2011). Biodegradable polymer scaffold for tissue engineering. Trends Biomater. Artif. Organs.

[133] Ehrlich H.P., Hunt T.K. (2012). Collagen Organization Critical Role in Wound Contraction. Adv. Wound Care.

[134] Kitamura M., Nakashima K., Kowashi Y., Fujii T., Shimauchi H., Sasano T., Furuuchi T., Fukuda M., Noguchi T., Shibutani T. (2008). Periodontal Tissue Regeneration Using Fibroblast Growth Factor -2: Randomized Controlled Phase II Clinical Trial. PLoS ONE.

[135] Kawaguchi H., Jingushi S., Izumi T., Fukunaga M., Matsushita T., Nakamura T., Mizuno K., Nakamura T., Nakamura K. (2007). Local application of recombinant human fibroblast growth factor-2 on bone repair: A dose–escalation prospective trial on patients with osteotomy. J. Orthop. Res..

[136] Wu J.-C., Huang W.-C., Chen Y.-C., Tu T.-H., Tsai Y.-A., Huang S.-F., Huang H.-C., Cheng H. (2011). Acidic fibroblast growth factor for repair of human spinal cord injury: A clinical trial. J. Neurosurg. Spine.

[137] Sanad R.A.-B., Abdel-Bar H.M. (2017). Chitosan–hyaluronic acid composite sponge scaffold enriched with Andrographolide-loaded lipid nanoparticles for enhanced wound healing. Carbohydr. Polym..

[138] Choi J.U., Lee S.W., Pangeni R., Byun Y., Yoon I.-S., Park J.W. (2017). Preparation and in vivo evaluation of cationic elastic liposomes comprising highly skin-permeable growth factors combined with hyaluronic acid for enhanced diabetic wound-healing therapy. Acta Biomater..

[139] Rabbani P., Zhou A., Borab Z.M., Frezzo J.A., Srivastava N., More H.T., Rifkin W., David J.A., Berens S.J., Chen R. (2017). Novel lipoproteoplex delivers Keap1 siRNA based gene therapy to accelerate diabetic wound healing. Biomaterials.

[140] Arantes V.T., Faraco A.A., Ferreira F.B., Oliveira C.A., Martins-Santos E., Cassini-Vieira P., Barcelos L.S., Ferreira L.A., Goulart G.A. (2020). Retinoic acid-loaded solid lipid nanoparticles surrounded by chitosan film support diabetic wound healing in in vivo study. Colloid. Sur. B.

[141] Palmer B.C., DeLouise L.A. (2020). Morphology-dependent titanium dioxide nanoparticle-induced keratinocyte toxicity and exacerbation of allergic contact dermatitis. HSOA J. Toxicol. Curr. Res..

[142] Palmer B.C., Phelan-Dickenson S.J., DeLouise L.A. (2019). Multi-walled carbon nanotube oxidation dependent keratinocyte cytotoxicity and skin inflammation. Part. Fibre Toxicol..

[143] Lai X., Wang M., Zhu Y., Feng X., Liang H., Wu J., Shao L. (2021). ZnO NPs delay the recovery of psoriasis-like skin lesions through promoting nuclear translocation of p-NFκB p65 and cysteine deficiency in keratinocytes. J. Hazard. Mater..

[144] Xiao H., Zhang H. (2020). Skin inflammation and psoriasis may be linked to exposure of ultrafine carbon particles. J. Environ. Sci..

[145] Wang M., Lai X., Shao L., Li L. (2018). Evaluation of immunoresponses and cytotoxicity from skin exposure to metallic nanoparticles. Int. J. Nanomed..

[146] Hashempour S., Ghanbarzadeh S., I Maibach H., Ghorbani M., Hamishehkar H. (2019). Skin toxicity of topically applied nanoparticles. Ther. Deliv..

[147] Pan Y., Paschoalino W.J., Blum A.S., Mauzeroll J. (2021). Recent Advances in Bio-Templated Metallic Nanomaterial Synthesis and Electrocatalytic Applications. ChemSusChem.

[148] Kang H., Buchman J.T., Rodriguez R.S., Ring H.L., He J., Bantz K.C., Haynes C.L. (2019). Stabilization of Silver and Gold Nanoparticles: Preservation and Improvement of Plasmonic Functionalities. Chem. Rev..

[149] Sooklert K., Nilyai S., Rojanathanes R., Jindatip D., Sae-Liang N., Kitkumthorn N., Mutirangura A., Sereemaspun A. (2019). N-acetylcysteine reverses the decrease of DNA methylation status caused by engineered gold, silicon, and chitosan nanoparticles. Int. J. Nanomed..

[150] Ali A., Suhail M., Mathew S., Shah M.A., Harakeh S.M., Ahmad S., Kazmi Z., Alhamdan M.A.R., Chaudhary A., Damanhouri G.A. (2016). Nanomaterial Induced Immune Responses and Cytotoxicity. J. Nanosci. Nanotechnol..

[151] Bakshi M.S. (2017). Nanotoxicity in Systemic Circulation and Wound Healing. Chem. Res. Toxicol..

[152] Hadrup N., Sharma A.K., Loeschner K. (2018). Toxicity of silver ions, metallic silver, and silver nanoparticle materials after in vivo dermal and mucosal surface exposure: A review. Regul. Toxicol. Pharmacol..

[153] Teixeira M.A., Paiva M.C., Amorim M.T.P., Felgueiras A.H.P. (2020). Electrospun Nanocomposites Containing Cellulose and Its Derivatives Modified with Specialized Biomolecules for an Enhanced Wound Healing. Nanomaterials.

[154] Xu L., Chu Z., Wang H., Cai L., Tu Z., Liu H., Zhu C., Shi H., Pan D., Pan J. (2019). Electrostatically Assembled Multilayered Films of Biopolymer Enhanced Nanocapsules for on-Demand Drug Release. ACS Appl. Bio Mater..

[155] Chen G., Chen Z., Wen D., Wang Z., Li H., Zeng Y., Dotti G., Wirz R.E., Gu Z. (2020). Transdermal cold atmospheric plasma-mediated immune checkpoint blockade therapy. Proc. Natl. Acad. Sci. USA.

[156] Xu L., Wang H., Chu Z., Cai L., Shi H., Zhu C., Pan D., Pan J., Fei X., Lei Y. (2020). Temperature-Responsive Multilayer Films of Micelle-Based Composites for Controlled Release of a Third-Generation EGFR Inhibitor. ACS Appl. Polym. Mater..

[157] Yu J., Wang J., Zhang Y., Chen G., Mao W., Ye Y., Gu Z. (2020). Glucose-responsive insulin patch for the regulation of blood glucose in mice and minipigs. Nat. Biomed. Eng..

[158] Abazari M., Ghaffari A., Rashidzadeh H., Momeni Badeleh S., Maleki Y. (2020). Current status and future outlook of nano-based systems for burn wound management. J. Biomed. Mater. Res. Part B Appl. Biomater..

[159] Dukhinova M.S., Prilepskii A.Y., Shtil A.A., Vinogradov V.V. (2019). Metal Oxide Nanoparticles in Therapeutic Regulation of Macrophage Functions. Nanomaterials.

[160] Janjic J.M., Gorantla V.S. (2017). Peripheral Nerve Nanoimaging: Monitoring Treatment and Regeneration. AAPS J..

[161] Ma Z., Li S., Wang H., Cheng W., Li Y., Pan L., Shi Y. (2019). Advanced electronic skin devices for healthcare applications. J. Mater. Chem. B.

[162] Asif M.H., Danielsson B., Willander M. (2015). ZnO Nanostructure-Based Intracellular Sensor. Sensors.

